# Improving efficiency in lung SAbR planning using integrated tools for X‐ray based adaptive radiotherapy

**DOI:** 10.1002/acm2.70195

**Published:** 2025-07-15

**Authors:** Justin Visak, Brien Washington, Chien‐Yi Liao, Sean Domal, David Parsons, Yuanyuan Zhang, Shahed Badiyan, Kenneth Westover, Mu‐Han Lin

**Affiliations:** ^1^ Department of Radiation Oncology University of Texas Southwestern Medical Center Dallas Texas USA; ^2^ Medical Artificial Intelligence and Automation Laboratory Department of Radiation Oncology University of Texas Southwestern Medical Center Dallas Texas USA

**Keywords:** adaptive radiotherapy, automation, lung‐SBRT, SAbR

## Abstract

**Purpose:**

To evaluate the feasibility of translating clinical lung stereotactic ablative radiotherapy (SAbR) templates from Ethos1.1 to Ethos2.0, leveraging new features to facilitate dose fall‐off and automate patient‐specific beam arrangement. This study aims to streamline planning processes and support broader adoption of online adaptive radiotherapy (ART) for lung SAbR.

**Methods:**

We selected fifteen patients previously treated with adaptive lung SAbR using the Ethos1.1 system, each receiving 40–60 Gy in 5 fractions. Plans were reoptimized in Ethos2.0 using identical parameters (rIMRT) to their clinical counterpart. To evaluate new integrated features, we utilized high‐fidelity (HF) mode with and without automatic treatment geometry selection (HF‐cIMRT, HF‐aIMRT/VMAT). These strategies were compared to assess the impact of Ethos2.0's new features on plan quality and efficiency using RTOG‐based metrics and enhanced plan deliverability analysis. Statistical significance was assessed using paired Student's *t*‐tests (*α* = 0.05).

**Results:**

All plans reoptimized in Ethos2.0 demonstrated acceptable plan quality. No statistically significant differences in maximum organ‐at‐risk doses were observed between evaluated strategies and the clinical plan. For complex cases, human‐selected beam geometry proved superior to automated geometry. HF‐enabled plans significantly reduced total monitor units, with HF‐aVMAT, HF‐cIMRT, and HF‐aIMRT reporting 3142.4 ± 997.4 (*p* < 0.001), 3401.8 ± 516.1 (*p* < 0.001), and 3225.6 ± 484.2 (*p* < 0.001) compared to clinical 5424.9 ± 1353.4. A trade‐off was observed in conformity index, which was 1.06 ± 0.08 (*p* = 0.006), 1.05 ± 0.06 (*p* = 0.003), and 1.03 ± 0.05 (*p* = 0.05) for HF‐aIMRT, HF‐cIMRT, and HF‐aVMAT plans compared to clinical 1.01 ± 0.03.

**Conclusion:**

Lung SAbR planning strategies can be effectively transitioned from Ethos1.1 to Ethos2.0, improving workflow efficiency with high‐fidelity mode and minor adjustments. Automated beam geometry tools enhance planner efficiency for both IMRT and VMAT. To address increased ART workload and staffing demands, leveraging integrated automation tools is essential. The planning strategies presented in this study are straightforward and reproducible for ART‐enabled clinics.

## 1 INTRODUCTION

Adaptive radiotherapy (ART) represents a rapidly evolving field with recent commercial availability of both online X‐ray based^1^, ^2^ and MR‐guided systems.^3^, ^4^ While offline ART has been discussed for over a decade,^5^, ^6^ online adaptive treatments have gained significant traction in recent years, introducing a paradigm shift in multidisciplinary radiotherapy practice.^7^ Lung radiation therapy, often concurrent with chemotherapy, is an established treatment option for patients with medically inoperable tumors.^8^, ^9^ Lung SAbR has emerged as a substantial area of interest for online adaptive therapy, as it enables customized plans for each fraction^10^ that potentially allow for reduced margin treatment and response‐based adaptation.^11^ This flexibility helps address inter‐fractional changes and enables personalized ultra‐fractionated stereotactic adaptive radiotherapy (PULSAR), which may optimize biological effects through elongated intervals between treatments.^12^ This also increases the importance of having a robust treatment planning method to ensure high‐quality online plans are generated against the potential changes between treatments.

Online adaptive radiotherapy presents several workflow^13^, ^14^ and technical challenges,^15^ including increased importance of reference planning and robustness considerations. In addition, the heightened treatment complexity suggests the need for alternative staffing models to accommodate increased technical demands. As the field matures, it undergoes constant workflow changes and technological advances, such as enhanced imaging capabilities^16^ and improved motion management.^17^ For example, the Ethos System was originally designed to support conventional adaptive treatments through its intelligent‐optimization‐engine (IOE), which mediates between the human planner and the Photon Optimizer to streamline conventionally‐fractionated treatment planning workflow.^2^ The IOE adds supplemental planning goals, such as dose‐flattening optimization, that are unseen to the user. To address this limitation, several robust dynamic optimization planning structures must be deployed to generate a SABR‐style plan, as reported in the literature. Gonzalez et al.^18^ addressed the importance of developing robust and readily shareable reference planning strategies for lung SAbR by utilizing dynamic hotspot tuning structures, inner and outer rings. Byrne et al. similarly reported that these dynamic structures are necessary to produce SAbR‐like plans.^19^ While these strategies generate high‐quality reference plans, they require manual adjustment on a per‐plan basis and increased planning time. The Ethos v1.1 system also requires manual determination of IMRT treatment geometry with limited VMAT use due to increased calculation times. These requirements necessitate enhanced planning knowledge and training to complete unique ART manual plan adjustments, making it challenging to quickly onboard or create cross‐coverage staffing models with existing dosimetric planning teams.

Recently, the Varian Ethos treatment planning system has undergone significant modifications, introducing features tailored for stereotactic ablative radiotherapy.^20^, ^21^ A notable enhancement is the high‐fidelity (HF) mode, designed to achieve steep dose gradients and deliver higher doses to the target.^22^ HF mode modifies IOE supervision by removing dose‐flattening secondary goals to allow for sharp dose‐gradients and higher dose in the target. To help combat overmodulation, HF‐mode increased fluence smoothing through larger MLC openings by deploying an increased complexity constraint to force higher fluence smoothing. Also, in conventional mode, two ring structures are created with specific distances and thicknesses from the target. High‐fidelity mode uses a single ring structure starting at 3 mm from the target with a thickness of 1.7 cm, and includes a larger normal tissue structure that aims to better control fall‐off at 2 cm from the target.^22^ These modifications facilitate dose gradients meeting national guidelines, aligning with other SAbR treatment planning strategies (for both IGRT and ART).^23^, ^24^ Specifically, This HF mode is of interest as it is recommended to produce high target heterogeneity to improve dose‐spillage outside of the target volume. This effectively will increase sparing of local healthy tissue/organs.^25^ Within the context of radiotherapy oncology group reports (RTOG)^8^, ^9^, dose‐spillage can be assessed using common surrogates such as the D2cm and gradient index. The D2cm is a simple way to assess the maximum dose any 2 cm away from the target but does not quantitively assess the “size” of intermediate dose spillage. Whereas the gradient index complements the D2cm by essentially quantifying the size of intermediate dose spillage around the target.

Additionally, the Ethos2.0 system introduces an automatic beam geometry optimization function capable of generating optimized IMRT and VMAT field geometries for esophageal and lung tumors.^22^ These advancements present an opportunity to improve lung SAbR plan quality while simultaneously reducing planner workload and enhancing delivery efficiency. In this study, we evaluate the performance of Ethos2.0's high‐fidelity mode and automated field geometry features, comparing them directly with the previous Ethos1.1 system for lung SAbR reference planning. We also assess whether clinical lung SAbR templates can be effectively transferred from Ethos1.1 to Ethos2.0. We hypothesize that these new features will streamline the planning process without compromising dosimetric quality. This supports broader adoption of online ART for lung SAbR

## 2 METHODS

### 2.1 Patient demographics and Ethos1.1 planning strategy

This study was conducted under Institutional Review Board approval (STU‐082013‐008). Fifteen patients with early‐stage NSCLC or lung metastases were treated with SAbR (40–60 Gy in five fractions) using the Ethos1.1 treatment system. See Table 1 for more information. Patient simulation utilized a 10‐phase 4D CT with compression applied if target motion exceeded 1cm during mini‐motion assessment. A maximum intensity projection (MIP) and average scan were generated for internal target volume (ITV) delineation and reference treatment planning. The ITV was expanded with an isotropic 5 mm margin to generate the planning target volume. Reference planning in Ethos1.1 utilized RTOG‐based dosimetric planning criteria with adapt‐recommended tuning structures to maximize online robustness. All plans employed 9–14 IMRT gantry angles primarily on the ipsilateral patient side. For each fraction, a CBCT was acquired, and all OAR/target contours were adjusted by the covering physician. Using the reference plan strategy, an adaptive plan was generated on daily contours for physician review and approval. Details of the online Ethos workflow are published elsewhere.^26^

**TABLE 1:** Average internal target volume and planning target volume including tumor location for all 15 patients.

Pt No.	ITV (cc)	PTV (cc)	Location
1	30.37	68.53	RUL/peripheral
2	7.74	26.13	LML/peripheral
3	2.05	8.94	LUL/peripheral
4	6.58	22.41	RUL/peripheral
5	19.13	47.16	RUL/central
6	55.66	104.55	LML/central
7	40.88	83.1	RML/central
8	3.2	13.44	LUL/peripheral
9	3.94	15.27	LLL/peripheral
10	22.73	60.16	LUL/central
11	11.03	32.96	LLL/peripheral
12	12.79	45.22	RUL/peripheral
13	6.59	21.43	RUL/peripheral
14	2.04	9.68	RUL/peripheral
15	2.35	10.97	RML/peripheral

### 2.2 Ethos2.0 treatment planning strategies

All patients were reoptimized using different strategies in Ethos2.0 to evaluate the novel integrated high‐fidelity and automatic beam geometry features. PTV coverage was normalized identically to the clinical plan (typically 95% of volume receives 100% of prescription dose). The planning template from Ethos1.1 was transferred to Ethos2.0 and reoptimized using clinical beam geometry (rIMRT plans). Our institutional practice incorporates a comprehensive set of control structures, including dose control rings at 3 mm, 1 and 2 cm, along with an inner dose shaping ring (PTV‐ITV + 1 mm) and a plan hotspot driving structure (ITV‐2 mm) to facilitate the SAbR dose distribution. Using this strategy as a baseline, the novel high‐fidelity mode was then enabled, and plans were iteratively reoptimized with a modified planning strategy to maximize plan quality with clinical beam geometry and automatic beam geometry selection (See 2.B.1. and 2.B.2 for more details). For comparison purposes, we generated five distinct treatment plans:

- **Clinical‐** Clinically treated plan using Ethos1.1 system.
- **rIMRT‐** Reoptimized plan in Ethos 2.0 using identical Ethos1.1 plan strategy and clinical IMRT beam geometry.
- **HF‐cIMRT‐** Reoptimized plan in Ethos 2.0 using modified Ethos2.0 plan strategy and clinical IMRT beam geometry
- **HF‐aIMRT‐** Reoptimized plan in Ethos 2.0 using modified Ethos2.0 plan strategy and automatic IMRT beam geometry.
- **HF‐aVMAT‐** Reoptimized plan in Ethos 2.0 using modified Ethos2.0 plan strategy and automatic VMAT beam geometry.

#### 2.2.1 High‐fidelity mode optimization

The HF‐enabled planning strategy was adjusted based on modified dose‐shaping features and prior planning knowledge. While the clinical plans typically deployed adaptive tuning structures (inner/outer rings, hotspot shaping) in higher priority levels (Priority‐1, Priority‐2) to drive SAbR‐like plan quality, HF mode enabled the removal of 3 and 10 mm rings and deprioritized the inner ring/hotspot driver to priority‐3, streamlining the planning process. All other aspects remained closely aligned with the clinical reference plan strategy. We systematically investigated alternative template configurations in early phase‐testing with high‐fidelity mode, however, for conciseness we present the most suitable strategy.

#### 2.2.2 Automated beam geometry selection

We evaluated the automatic beam geometry function for both IMRT and VMAT geometry against clinically treated plans. Using the streamlined high‐fidelity planning strategy, we generated one IMRT and one VMAT plan (HF‐aIMRT, HF‐aVMAT). Beam and arc utilization frequency was recorded in 30° sector increments to compare overlap between clinical and automatic selections. We also assessed the average number of beams used clinically versus automatically for IMRT. No automated plan geometry was adjusted prior to plan optimization and was directly used for plan generation.

### 2.3 Dosimetric and plan deliverability metrics

All plans were evaluated using RTOG‐based metrics, including PTV hotspot, conformity index, gradient index, and D2cm measurements. We recorded both maximum and volumetric doses to organs at risk (OARs), focusing on maximum doses for this analysis. The assessment included doses to spinal cord, bronchus, trachea, heart, and esophagus. Lung dose was monitored using V13.5Gy per institutional guidelines. To assess plan deliverability and online adaptation feasibility, we recorded calculation time with 2 mm grid enabled for both HF‐enabled and HF‐disabled scenarios. Plan quality metrics were comprehensively documented through total monitor units (MU), modulation complexity score, SAS10, penumbra width, and edge penalty measurements to establish delivery metrics baselines. We conducted independent Monte Carlo‐calculation based IMRT quality assurance with a gamma tolerance of 3%/2 mm to assess deliverability. Statistical comparisons between all Ethos2.0 plans and clinical plans were performed using paired Student's t‐tests.

## 3 RESULTS

### 3.1 RTOG‐based target metrics

**FIGURE 1:**
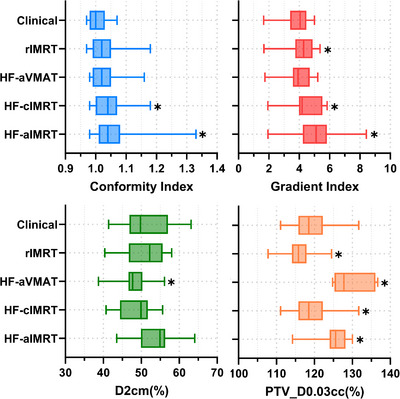
RTOG‐based plan metrics including PTV hotspot. Conformity index, gradient index, D2cm(%) and PTV hotspot. Statistical significance to clinical plan is denoted by *. Note that on average all plans demonstrate acceptable CI, GI and D2cm and plan hotspot. HF‐enabled plans typically generate a higher plan hotspot while maintaining sharp‐dose fall off where the automatic VMAT plan demonstrates on average the sharpest fall‐off with increased hotspot.

All treatment plans achieved acceptable quality standards according to RTOG criteria. Conformity index analysis revealed modest differences between approaches: clinical plans averaged 1.01 ± 0.03, while HF‐enabled plans showed slight increases to 1.06 ± 0.08 (*p* = 0.006) for HF‐aIMRT, 1.05 ± 0.06 (*p* = 0.003) for HF‐cIMRT, and 1.03 ± 0.05 (*p* = 0.05) for HF‐aVMAT. The reoptimized rIMRT plans demonstrated similar results with a CI of 1.03 ± 0.05 (*p* = 0.05). Dose distribution characteristics varied among plan types. As shown in Figure 1, gradient index increased for all Ethos2.0 plans compared to clinical values, though only HF‐aVMAT cases maintained statistical equivalence. The maximum dose at 2 cm (D2cm) showed improvement across some of the new planning approaches compared to the clinical value of 51.1 ± 6.0%. The greatest improvement was observed with HF‐aVMAT at 47.7 ± 3.8% (*p* = 0.01), followed by HF‐cIMRT at 48.9 ± 3.9% (*p* = 0.09) and rIMRT at 50.7 ± 5.0% (*p* = n.s.). Target dose heterogeneity showed distinct patterns between planning approaches. As illustrated in Figure 1, PTV hotspot decreased from 119.0 ± 4.9% in clinical plans to 116.0 ± 3.7% (*p* = 0.01) in rIMRT cases. In contrast, HF‐enabled plans demonstrated systematically higher hotspots: 125.5 ± 3.8% (*p* = 0.001) for HF‐aIMRT, 127.4 ± 3.0% (*p* < 0.001) for HF‐cIMRT, and 129.6 ± 4.5% (*p* < 0.001) for HF‐aVMAT.

### 3.2 OAR sparing

Maximum organ‐at‐risk doses for all 15 patients demonstrated consistency across treatment approaches, as shown in Table 2. None of the plans showed statistically significant differences in maximum doses to the spinal cord, esophagus, heart, trachea, or bronchus compared to clinically treated cases. The lung dose metric V13.5Gy, however, showed small but statistically significant increases across all plan types.

**TABLE 2:** Maximum dose to organs‐at‐risk and V13.5Gy in lung‐ITV for all 15 patients and plan type.

Plan type	Spinal cord (cGy)	Esophagus (cGy)	Heart (cGy)	Trachea (cGy)	Bronchus (cGy)	V13.5Gy lungs‐ITV (cc)
HF‐aIMRT	1025.3 ± 501.2	1478.1 ± 930.9	759.6 ± 1042.4	1418.5 ± 1238.6	1399.8 ± 1430.6	263.5 ± 132.5*
HF‐cIMRT	945.2 ± 315.2	1454.9 ± 962.2	700.1 ± 1006	1442.9 ± 1188.4	1330.2 ± 1417.2	262.8 ± 129.9*
HF‐aVMAT	927.9 ± 354.9	1370.6 ± 944.8	730.2 ± 1068.3	1410.2 ± 1231.7	1330.3 ± 1465.8	233.4 ± 121
rIMRT	955.5 ± 331.1	1456.2 ± 874.2	743.2 ± 1081.9	1489.9 ± 1214.9	1349.2 ± 1431.0	239.1 ± 118.3*
Clinical	902.2 ± 317.7	1462.5 ± 885.5	758.7 ± 1128.2	1463.2 ± 1167.6	1364.1 ± 1387.7	225.4 ± 116

No statistically significant differences were observed compared to clinically treated cases for maximum dose whereas statistical significant differences were observed in Lung‐ITV constraints. *Denotes statistical significance.

### 3.3 Automated beam selection quality

**FIGURE 2:**
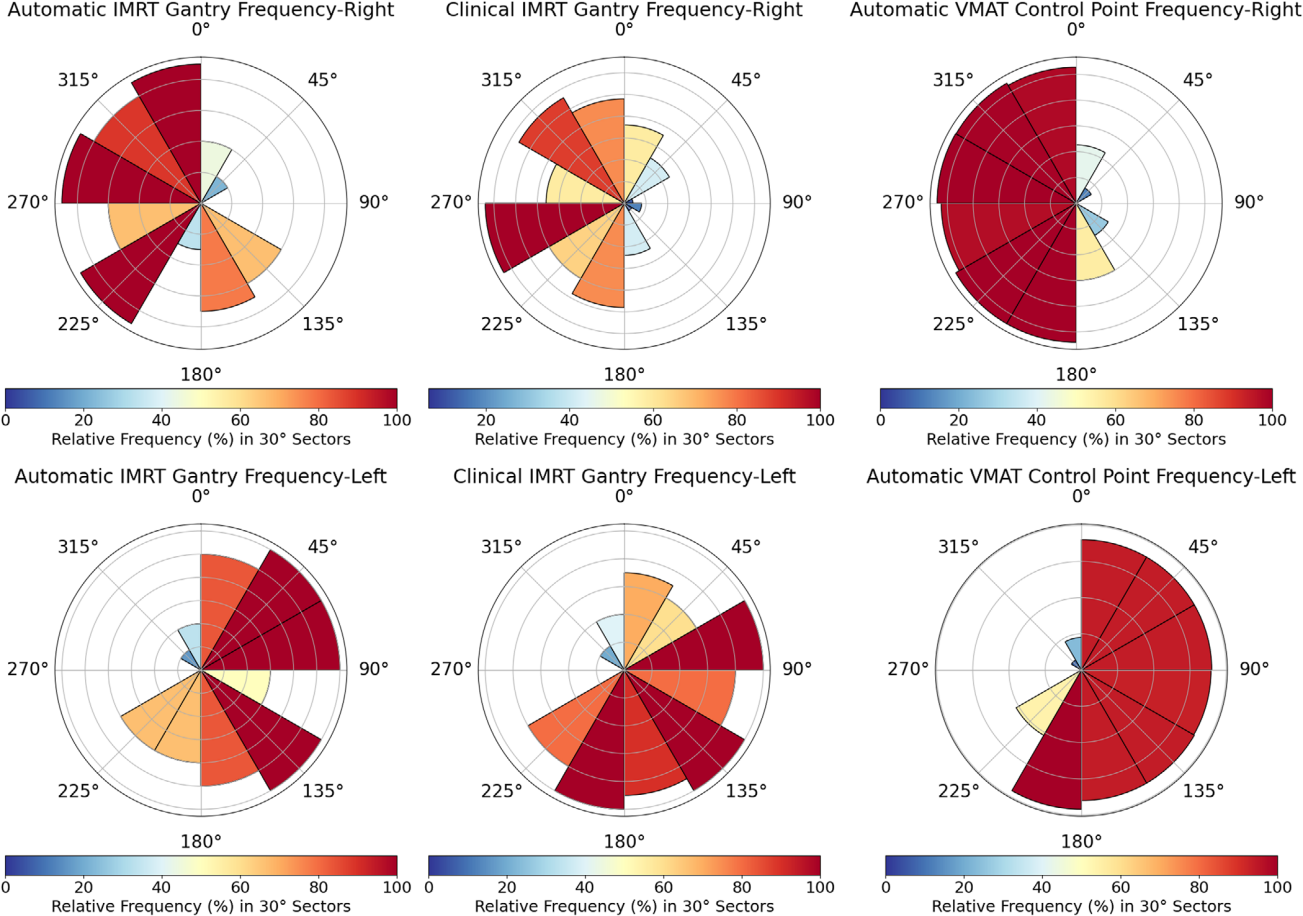
Comparative analysis of gantry angle frequencies in IMRT and control point in VMAT techniques for clinical and automatic algorithm. Polar histograms of angle utilization for all 15 patients. Human‐selected angles prefer to spread out beams to contralateral side to boost conformity whereas the algorithm‐selected angles tend to cluster on the ipsilateral treatment side. This difference highlights the varying strategies between human intuition and algorithmic optimization.

Analysis of gantry angle utilization revealed distinct patterns between human‐selected and algorithm‐selected beam arrangements as shown in Figure 2. The automatic IMRT algorithm consistently utilized 7 beams across all cases, compared to an average of 12 beams (range: 9–14) in clinical plans. Human planners typically selected a broader range of sectors to maximize plan quality and dose conformity, whereas the aIMRT algorithm showed a strong preference for ipsilateral arrangements. For example, in the 0–30° sector for right‐sided tumors, the algorithm deployed a beam 40% of the time compared to 60% utilization by human planners. Similarly, for left‐sided tumors in comparable sectors (e.g., 330–360°), algorithm utilization was 35% compared to 65% for human planners.

The aVMAT approach typically employed 2–3 partial arcs, with ipsilateral sectors utilized over 90% of the time. Contralateral sector utilization dropped as low as 10% in sector 2 (30–60°) for right‐sided tumors and sector 11 (300–330°) for left‐sided cases. Sectors adjacent to the primary tumor location showed consistently high utilization rates of 85%–95% in both automatic IMRT and VMAT plans, whereas sectors beyond 90° from the primary tumor location showed utilization rates below 30% in automatic plans compared to 45%–55% in human‐selected arrangements.

### 3.4 Plan deliverability and generation time

**FIGURE 3:**
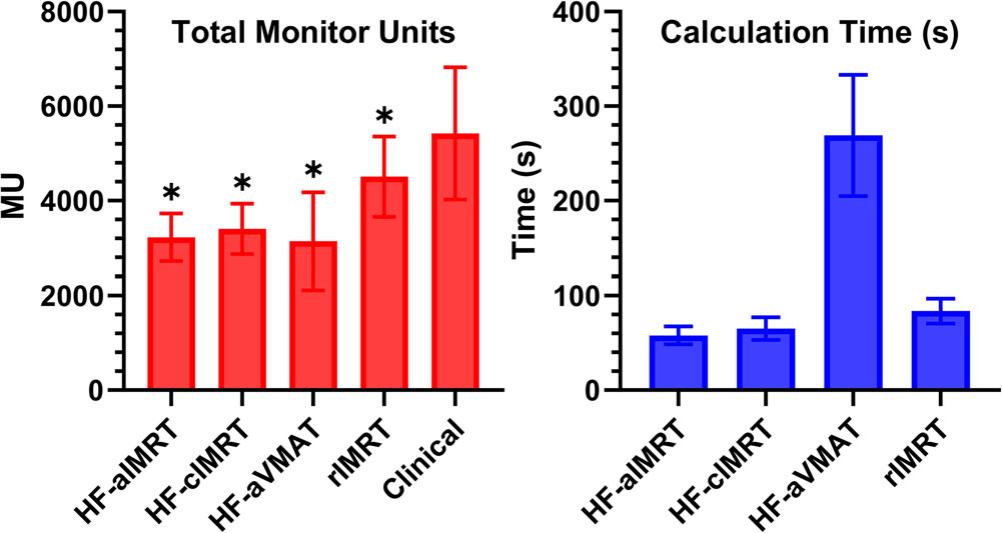
Total monitor units and reference plan generation time for all 15 patients. Total MU reported for all patients including clinically treated reference plans. Calculation time bar does not include calculation time for clinical cases, as the emulator and clinical system have different graphical processing unit speeds and is not an appropriate comparison. Note that all Ethos2.0 optimized plans generated lower MU with similar calculation times (not including HF‐aVMAT). The VMAT‐generated cases produced the lowest MUs at the cost of calculation time.

We assessed plan deliverability for all 15 patients and plan generation time for Ethos2.0 plans only, as the emulator system's different GPU configuration made clinical timing comparisons inappropriate. As shown in Figure 3, all Ethos2.0 optimized plans achieved significant reductions in monitor units compared to the clinical average of 5424.9 ± 1353.4 MU. HF‐enabled plans demonstrated the largest reductions: HF‐aVMAT at 3142.4 ± 997.4 MU (*p* < 0.001), HF‐aIMRT at 3225.6 ± 484.2 MU (*p* < 0.001), and HF‐cIMRT at 3401.8 ± 516.1 MU (*p* < 0.001). The reoptimized rIMRT plans showed a more modest improvement at 4508.5 ± 820.9 MU (*p* = 0.02). Optimization times varied by delivery technique, with standard Ethos2.0 IMRT plans completing in less than 90 s and HF‐aVMAT plans requiring 269.1 ± 62 s. Plan complexity measurements showed consistent improvements across newer planning approaches. The modulation complexity score (1‐MCS) decreased from the clinical value of 0.78 ± 0.04, indicating reduced complexity in rIMRT (0.76 ± 0.03, p = n.s.), HF‐aVMAT (0.72 ± 0.07, *p* = 0.01), HF‐cIMRT (0.69 ± 0.03, *p* < 0.001), and HF‐aIMRT (0.67 ± 0.04, *p* < 0.001). Analysis of small field openings (SAS10%) showed significant reductions across all Ethos2.0 plans compared to the clinical average of 39.9% ± 11.8%. HF‐aIMRT achieved the lowest percentage at 20.7% ± 4.2% (*p* < 0.001), followed by HF‐cIMRT at 21.9% ± 3.3% (*p* < 0.001), HF‐aVMAT at 29.6 ± 10% (*p* < 0.02), and rIMRT at 31.3 ± 5.8% (*p* = 0.004). All plans achieved IMRT QA passing rates exceeding 99%.

### 3.5 Example patient

**FIGURE 4:**
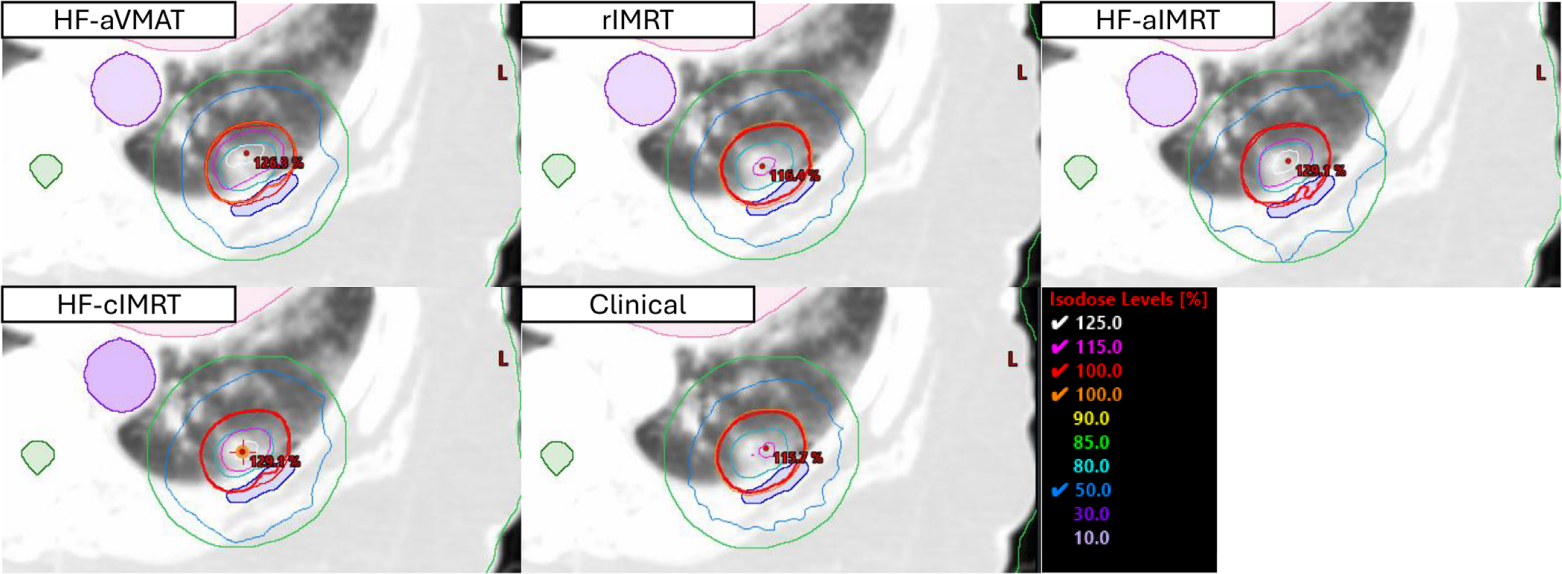
Axial views of example patient for each strategy with isodose lines. Corresponding axial slices with isodose lines with identical isodose levels are shown. Additionally, the heart (pink), aorta (purpose), spinal cord (dark green), d2cm (green), ITV (light blue) and PTV (red) are shown. Note that all HF plans feature a larger central 125% hotspot. The 50% IDL is qualitatively similar across all plans, except for the automatically generated IMRT case, where the shape suggests more beams were necessary but still acceptable.

To illustrate the cohort findings at an individual level, we present a representative case with an ITV of 11cc and PTV of 33cc where the patient received 60 Gy in five fractions in Figure 4. OAR doses showed minimal variation from clinical cases across all plan types. Two key distinctions emerged in plan characteristics: plan hotspot and intermediate dose fall‐off variations between planning techniques. The clinical plan achieved a PTV hotspot of 115.8%, while the reoptimized rIMRT reached 116.2%, demonstrating that maintaining similar planning strategies produces closely matched results. HF‐enabled modes generated higher hotspots of 129% for HF‐aIMRT and 128% for both HF‐aVMAT and HF‐cIMRT cases, without compromising OAR sparing. The D2cm remained quantitatively similar across all cases, ranging from 46.5% to 49.9% of the prescription dose. The HF‐aIMRT plan showed distinct characteristics in intermediate dose distribution, likely due to reduced beam numbers, impacting intermediate dose fall‐off and target conformity. While meeting acceptable quality standards, the 50% isodose line showed notable differences compared to other techniques. Monitor unit reduction was observed across all plans compared to the clinical reference plan (8621.2 MU), with HF‐aVMAT requiring 2703.2 MU, HF‐aIMRT 3636.0 MU, HF‐cIMRT 3676.4 MU, and rIMRT 4855.1 MU. The conformity index showed corresponding variations, with the clinical plan achieving 0.98 compared to 1.0 for rIMRT, 1.04 for HF‐cIMRT, 1.06 for HF‐aVMAT, and 1.08 for HF‐aIMRT, all remaining within RTOG protocol compliance.

## 4 DISCUSSION

The integration of high‐fidelity mode in the Ethos2.0 system shows promise for streamlining lung SAbR planning in adaptive therapy. Our findings demonstrate that existing Ethos1.1 planning strategies transfer effectively to Ethos2.0, with IOE2.0 updates contributing to reduced plan hotspots. High‐fidelity mode, with minor adjustments to existing strategies, enables a more streamlined workflow while maintaining dosimetric quality. Notably, higher planning hotspots can be achieved with reduced manual input, simplifying the planning process without compromising quality. The automated beam geometry tool shows potential for enhancing planner efficiency, particularly with VMAT plans, despite longer computation times. Additionally, HF plans showed a reduction in monitor units (MU) and may help support a reduction in interplay effects for institutions with a preference for less modulation.

A caveat of these new workflows is that automated IMRT arrangements require careful consideration due to potential intermediate dose spill compromising plan quality. As stated in Hoffman et al., there is a predictable relationship between GI and PTV size < 85cc that can be used to assess plan quality beyond the RTOG‐provided deviation scoring for VMAT plans.^27^ In this work, we recorded larger than estimated GI for select cases (e.g., larger value for HF‐aIMRT). This is likely due to the selection of inferior IMRT geometry whereas in Hoffman's study it can be assumed a clinically reasonable geometry was always utilized. To address this limitation, we conducted additional testing with five patients who showed suboptimal results using the default automatic seven‐field arrangement (default‐auto7B). We developed an enhanced approach using the beam angle geometry optimizer (BGO), increasing the field number from 7 to 10 (enhanced‐auto10). This was accomplished by creating a temporary intent with a 25 Gy per fraction dose, which triggers the Ethos2.0 system to automatically increase field numbers in high‐fidelity mode. After extracting this geometry, we reimported it into the original planning intent for comparison. The enhanced‐auto10B plans demonstrated superior intermediate dose‐fall characteristics compared to default‐auto7B plans. D2cm values improved from 54.8 ± 2.7% to 49.9 ± 2.4%, while gradient index values improved from 5.6 ± 1.5 to 4.6 ± 0.7. These improvements were achieved without increasing average monitor units (3322.1 ± 630 for default‐auto7B versus 3410.9 ± 686 for enhanced‐auto10B) and maintained consistent organ‐at‐risk sparing.

**FIGURE 5:**
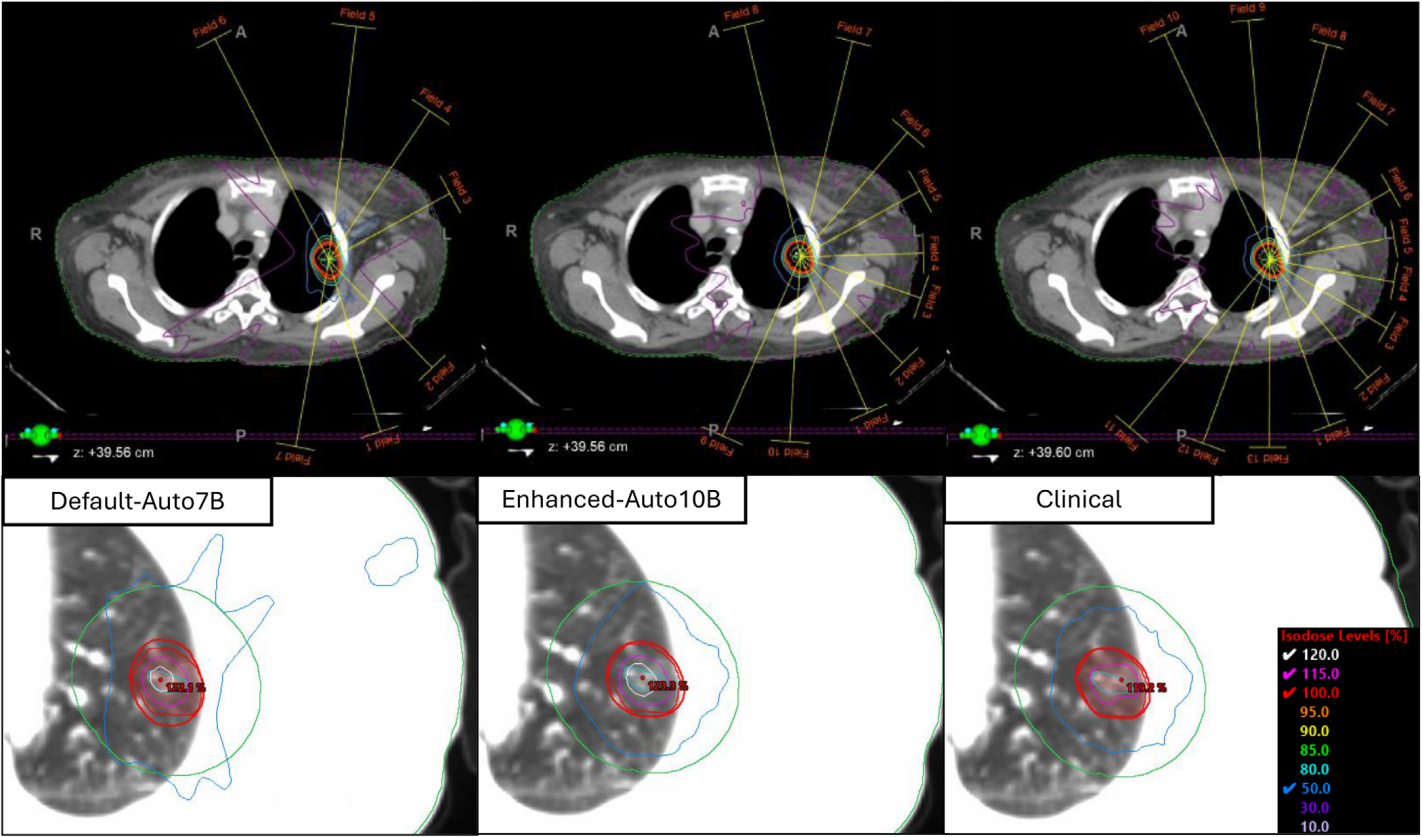
Example patient automatic geometry selection versus clinical including corresponding axial isodose lines. Similar arc sector usages are observed in all plans, however, the enhanced‐auto10B plan appears to more closely mimic the human selected field geometry. It is evident that in the corresponding axial isodose lines, this simple enhancement helps greatly improve the 50% RX spill into normal tissues.

Figure 5 illustrates these improvements in a representative patient case. While the enhanced approach achieves better plan quality, it requires approximately 15 additional minutes of planning time. This trade‐off between automation and manual refinement suggests that human expertise in treatment geometry optimization remains essential for optimal outcomes. Our study represents the first systematic evaluation of high‐fidelity mode for lung SAbR adaptive reference planning. Building on established planning principles, we demonstrate its potential to streamline the transition from conventional to adaptive planning workflows.

**FIGURE 6:**
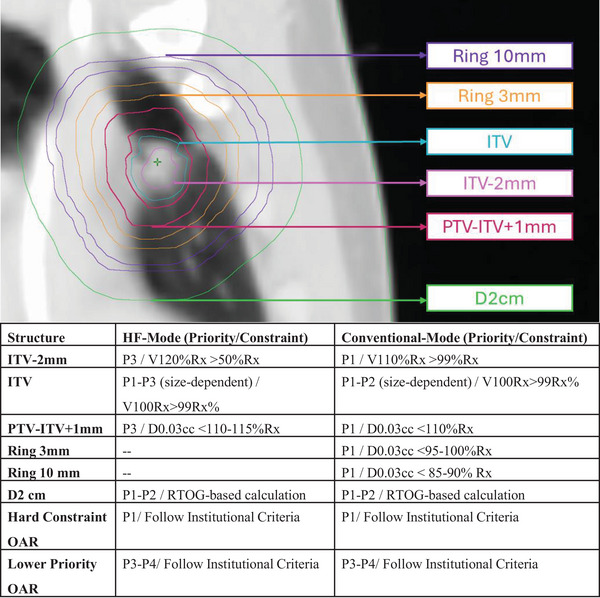
Visual representation with general optimization layering for dose fall‐off shaping. Note the reduction of total number of constraints and priority level for HF‐enabled mode vs conventional IOE. Additionally, ITV hotspot shaping constraints are weakened compared to conventional as HF natively achieves higher values.

The planning strategies we developed are straightforward and reproducible, making them suitable for all ART‐enabled clinics, both current and future. It has been previously shown that conventional skillsets do not directly translate to adaptive radiotherapy and additional training/onboarding is required.^7^ Having a simple‐to‐follow planning workflow, as presented in this manuscript, may support easier transitions by providing a structured approach that can be consistently applied across different scenarios. This can help streamline the onboarding process, reduce the learning curve for new team members, and ensure the necessary skills and knowledge is utilized to effectively implement adaptive lung SAbR. Additionally, a standardized workflow can facilitate better communication and collaboration within the team. This accessibility is particularly important as institutions face increasing workload demands from adaptive radiotherapy programs, creating significant staffing challenges. Viscariello et al.^28^ quantified this challenge through effort assessments, demonstrating the need for modified staffing models to handle the complexity of adaptive workflows. One way to address these staffing pressures is through increased automation. The original Ethos1.1 system, not specifically designed for SAbR treatments, required multiple optimization structures for robust dose shaping. This complexity made training and cross‐coverage particularly challenging. Our study addresses these limitations by providing reproducible planning strategies with clearer optimization priorities, as illustrated in Figure 6. Institutional practices may vary, but in general, we recommend placing any hard constraints at the top of the priority order. Constraints that are further away should be positioned below the target shaping structures, as this will ultimately help control the dose in these areas as well. For example, the ribs should be prioritized based on their proximity to the target and institutional practice. The streamlined approach we present is especially valuable as adaptive radiotherapy continues to evolve. Since reference planning forms the foundation of successful online treatment, institutions need workflows that can adapt to technological advances while maintaining efficiency and quality

**FIGURE 7:**
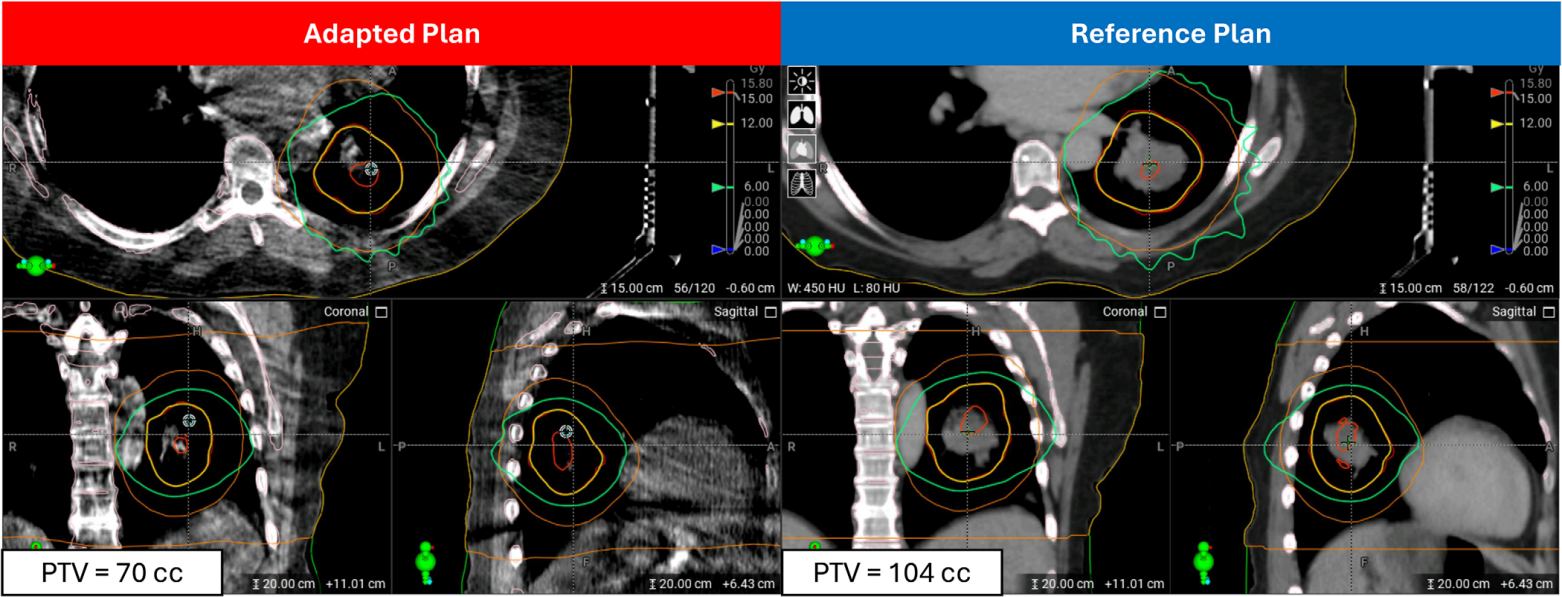
Adaptive versus scheduled plan comparison for HF‐cIMRT case. To demonstrate the robustness of our methods, we selected one patient's final fraction who received personalized ultra‐fractionated stereotactic ablative RT (PULSAR) for online emulation. The significantly smaller PTV highlights the high‐fidelity mode's ability to handle large changes. This figure shows the dose cloud shrinking compared to the reference plan, reducing OAR and normal lung doses, with notable gradient improvement near the heart.

Our study has two key limitations. First, while we have not yet validated these planning strategies in an online adaptive setting, their foundation in established literature and clinical experience suggests they should perform well for straightforward cases. For example, Gonzalez et al. reports on the robustness of adaptive planning strategies in an online setting and notes there was high compliance between the adaptive plan and reference plan for both OAR and target coverage.^18^ Additionally, it has been documented in literature there is not significant change in daily anatomy during a short course of (<5 fractions) by Regnery et al. In their study, the PTV size during MR‐guided RT change during treatment on average 0.4cc which supports the assumption that the planning strategy will be valid online.^29^ However, to demonstrate that our proposed methods are robust as indicated by literature, we selected one patient's final fraction who received personalized ultra‐fractionated stereotactic ablative RT (PULSAR)^30^ for online emulation. This PTV was significantly smaller than the reference planning structure and highlights the proposed high‐fidelity mode is robust to large change. Figure 7 shows that the dose cloud significantly shrank compared to the reference plan which reduced OAR dose and normal lung dose by robustly handling a 34cc reduction in PTV size. While this target size change is significant, we typically utilize a 45 min timeslot for the adaptive workflow for lung SAbR patients. Another limitation of this study is we did not explore knowledge‐based planning (KBP) models, which are integrated into Ethos2.0 and might further improve lung SAbR planning efficiency. Despite these limitations, our study makes important contributions to the field. We provide a comprehensive evaluation of Ethos2.0's impact on lung SAbR plan quality and demonstrate optimal approaches for leveraging both high‐fidelity mode and automated beam geometry features. Notably, because our method does not depend on institution‐specific models like KBP, it can be readily adopted by other clinics without modification.

## 5 CONCLUSION

This investigation demonstrates the successful transition of lung SAbR planning strategies from Ethos1.1 to Ethos2.0, achieving improved workflow efficiency through high‐fidelity mode implementation with minimal modifications. The automated beam geometry tools enhance planner efficiency for both IMRT and VMAT, though careful consideration is needed for automated IMRT implementation. As institutions face increasing ART workload and staffing demands, the integration of automated planning tools becomes essential. The planning strategies presented here offer straightforward, reproducible solutions for ART‐enabled clinics while emphasizing the importance of developing workflows that can adapt to continuing technological evolution in the field.

## AUTHOR CONTRIBUTIONS

J.V. and M.L. designed the study. J.V., B.W. and M.L. performed replanning of clinical cases. S.D., C.Y. and B.W. performed data collection, software design or statistics for this project. J.V., M.L. drafted the initial version of manuscript. Y.Z., S.B., K.W. provided clinical supervision and input to the project. All authors revised and approved the final manuscript.

## ETHICS STATEMENT

Institutional Review Board approval was obtained for the use of all patient data included in this study (STU 082013‐008).

## CONFLICT OF INTEREST STATEMENT

No conflicts of interest.

## Data Availability

The data supporting the findings of this study are available from the corresponding author upon reasonable request.
